# A novel antituberculosis agent exhibits potent clinical efficacy and good safety profile: an open-label, randomized-controlled, multicenter, phase 2a trial

**DOI:** 10.1038/s41392-025-02517-z

**Published:** 2025-12-30

**Authors:** Naihui Chu, Wenjuan Nie, Juan Du, Manni Wang, Liping Ma, Qingfeng Wang, Jun Wang, Xiaomeng Hu, Jin Wu, Yu Lu, Mailing Huang, Yuquan Wei, Zhenling Wang, Jian-Qing He, Zhenyu Ding, Xiawei Wei

**Affiliations:** 1 Department of Tuberculosis, Beijing Chest Hospital, Capital Medical University, Beijing Tuberculosis and Thoracic Tumor Institute, Beijing, China; 2 Wuhan Pulmonary Hospital (Wuhan Institute for Tuberculosis Control), Wuhan, Hubei China; 3 Department of Biotherapy, Cancer Center and State Key Laboratory of Biotherapy, National Clinical Research Center for Geriatrics, West China Hospital, Sichuan University, Chengdu, Sichuan China; 4 Jumbo Drug Bank Co., Ltd, Chengdu, China; 5 Department of Pulmonary and Critical Care Medicine, State Key Laboratory of Respiratory Health and Multimorbidity, West China Hospital, Sichuan University, Chengdu, China

**Keywords:** Infectious diseases, Translational research

## Abstract

Tuberculosis (TB) is a contagious disease that threatens human health worldwide. Combination chemotherapy is usually recommended for this disease. Recently, 2 nitroimidazole-based agents, namely, delamanid and pretomanid, have been approved by regulatory agencies. JDB0131 is a novel, structurally optimized third-generation nitroimidazole antituberculosis agent that incorporates the advantages of earlier compounds. This multicenter, prospective, randomized phase 2a trial was conducted to evaluate its efficacy and safety in patients with tuberculosis (NCT06224036). In total, 52 patients with newly diagnosed TB were recruited. JDB0131 was tested in a dose escalation manner (cohort 1: 100 mg bid, cohort 2: 200 mg qd, and cohort 3: 200 mg bid). For comparison, delamanid (100 mg bid) and classic fixed-dose combination (FDC) regimens were included as controls. The primary endpoint was logarithmic changes in the number of colony formation units (CFUs) in the solid media culture of sputum TB (log10 CFU). The early bactericidal activity (EBA) of JDB0131 was better than that of delamanid. During the time interval between days 0 and 14, JDB0131 at a dose of 200 mg bid (cohort 3) showed superior efficacy over delamanid. At the end of drug intervention (day 14), JDB0131 (all 3 dose levels) achieved superior time to positivity (TTP) over delamanid. Ninety-one adverse events (AEs), including no serious AEs, were attributed to JDB0131 in 30 patients. This trial identified a promising new drug for the increasing TB burden worldwide.

## Introduction

Tuberculosis (TB), a contagious disease, poses a critical threat to human health worldwide. It is caused by the bacillus *Mycobacterium tuberculosis*. According to the latest WHO Global TB Report, more than 10 million people develop active TB annually, and more than 1 million deaths are attributed to the disease, making it one of the leading infectious killers worldwide.^1^ The burden is particularly severe in low- and middle-income countries, where TB often coexists with HIV and is malnourished, further complicating treatment outcomes.^2^ For the treatment of this disease, a combination chemotherapy regimen of rifampicin, isoniazid, pyrazinamide, and ethambutol is recommended. The treatment is both lengthy and toxic.

Moreover, multidrug-resistant TB (MDR-TB) often develops either de novo (3–4%) or as a result of treatment failure (18–21%). The treatment for MDR-TB is still challenging. The success rate for MDR-TB therapy remains disappointingly low, usually below 60%, and many regimens extend over 18 months or longer.^3^ The current treatment paradigm requires updates, and agents with more potent anti-TB effects and safer profiles are urgently needed.

In recent years, two nitroimidazole-based agents, namely, delamanid and pretomanid, have been approved by regulatory agencies for MDR-TB treatment.^4–7^ These approvals represented landmark advances, marking the first introduction of new chemical classes for TB therapy in recent decades.^8^ Both agents have demonstrated clinically meaningful benefits, particularly when integrated into novel short-course regimens.^9^ Both suffer from poor aqueous solubility,^10^ together with nonlinear dose effects. Both compounds prolong the QT interval,^11–14^ which is notorious for their potential lethal effects in the clinic. This safety concern not only limits their clinical use in patients with preexisting cardiac risk factors but also necessitates intensive ECG monitoring during therapy, complicating implementation in high-burden settings.^15^

We developed a novel anti-TB agent, JDB0131, by modifying the structure of delamanid.^16^ This new compound exhibited excellent in vitro and in vivo antimycobacterial activities similar to those of delamanid. It also displays superior pharmacokinetic (PK) properties, excellent oral absorption, dose-dependent exposure, and no apparent accumulation in animals.^16^ Importantly, no QT interval prolongation was observed in our 39-week Good Laboratory Practice (GLP) toxicology studies in dogs.^16^ Taken together, these findings suggest that JDB0131 may retain the potent antimycobacterial activity of nitroimidazoles while overcoming key pharmacological and safety barriers that have limited the broader use of its predecessors. We strongly propose the agent JDB0131 as a clinical candidate. Its development aligns with the global imperative to identify safer, shorter, and more effective regimens for MDR-TB.^17^ To explore the clinical activities and safety profile, we conducted an open-label, random-controlled, multicenter, phase 2a trial (NCT06224036). Here, we report the main results of this trial.

## Results

### Demographic features

**Fig. 1:**
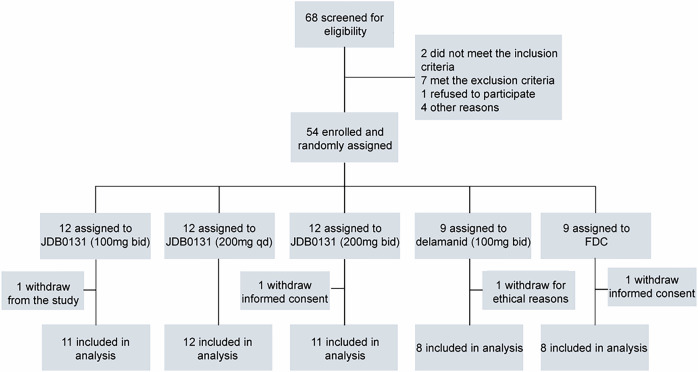
Flow of participants. Four participants did not complete the 14-day period of drug intake. One patient was randomized to JDB0131 100 mg bid, and one patient was randomized to delamanid and withdrawn. One patient randomized to JDB0131 200 mg bid withdrew consent

To characterize the study population, we summarized the enrollment flow and baseline demographics of all randomized patients. From November 2023 to August 2024, 68 patients were screened, and 54 patients were recruited (Fig. 1). Eligible patients were randomized into 5 cohorts. Four patients discontinued the study after randomization: two withdrew consent voluntarily, one was withdrawn by the investigator for the participant’s best interest, and one was withdrawn owing to ethical considerations (Fig. 1). The demographic features of these patients are summarized in Table 1. Patient enrollment was based on sputum smear positivity at screening, while sputum culture was not employed as an inclusion criterion. Rifampin resistance, an exclusion criterion, was confirmed via the Xpert® MTB/RIF Ultra assay, which is independent of culture results. All patients were smear positive and treatment naive, and a few (*n* = 5, 9%) were culture positive. These smear-positive but culture-negative results are well recognized in tuberculosis diagnosis and often reflect nonviable organisms following treatment or the technical limitations of culture methods.^18–20^ Nineteen patients had comorbidities, most commonly pulmonary infections and diabetes mellitus. Accordingly, 10 patients were taking concurrent antibiotics, and 3 were taking oral antidiabetics (OADs). Forty-nine patients (89%) had abnormal radiographic manifestations on baseline chest computed tomography, 38 of whom had cavitation.

**Table 1:** Demographic, anthropometric, and diagnostic features of the participants

Characteristic	JDB0131 (100 mg bid)	JDB0131 (200 mg qd)	JDB0131 (200 mg bid)	Delamanid (100 mg bid)	FDC
No. of participants	12	12	12	9	9
Age (median, minimum-maximum)	47.0 (22.0–64.0)	43.5 (24.0–65.0)	43.5 (20.0–62.0)	53.0 (19.0–63.0)	42.0 (20.0–61.0)
Sex					
Male, *n* (%)	10 (83.33)	11 (91.67)	9 (75.00)	8 (88.89)	6 (66.67)
Female, *n* (%)	2 (16.67)	1 (8.33)	3 (25.00)	1 (11.11)	3 (33.33)
Weight, mean (kg ± SD)	62.05 ± 6.27	61.74 ± 9.72	55.93 ± 11.96	58.00 ± 8.08	62.54 ± 12.02
BMI, mean (kg/m^2^ ± SD)	21.86 ± 2.97	20.87 ± 2.61	20.10 ± 2.94	19.25 ± 2.33	21.65 ± 2.67
Baseline sputum CFU (log CFU/ml sputum ± SD)	5.41 ± 1.28	5.87 ± 1.01	5.54 ± 1.87	4.41 ± 2.07	5.46 ± 1.55
Baseline TTP (h±SD)	228.86 ± 82.54	212.33 ± 82.51	192.50 ± 76.72	245.00 ± 96.16	326.00 ± 309.93

### Early bactericidal activity (EBA)

**Fig. 2:**
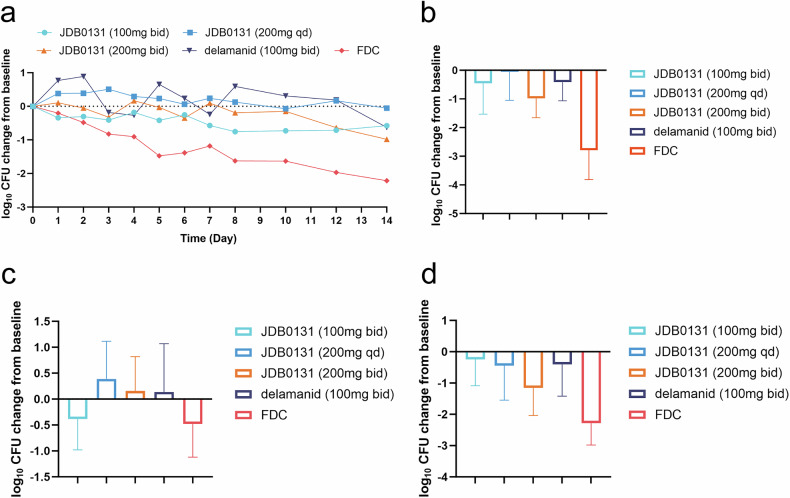
The logarithmic change in the number of colony-forming units (log_10_CFU) in solid cultures of *M. tuberculosis* in sputum. **a** Changes in log10CFU from baseline from Day 1 to Day 14. Single data points represent the mean values of individual treatment groups. **b** The bar chart shows the log_10_CFU changes from baseline for each group from Day 1 to Day 14. **c** The bar chart shows the log_10_CFU changes from baseline for each group from Day 1 to Day 2. **d** The bar chart shows the log_10_CFU changes from baseline for each group from Day 2 to Day 14

We assessed early EBA over 14 days to evaluate the initial microbiological response across treatment cohorts. Forty-seven patients received full-course treatment per protocol (*n* = 9 in cohort 1, *n* = 12 in cohort 2, *n* = 10 in cohort 3, *n* = 8 in the delamanid cohort, and *n* = 8 in the fixed-dose combination (FDC) cohort). At baseline (day 0), the TB burden in sputum (log_10_ CFU/ml) was roughly equally distributed among each cohort, except for a slightly lower TB burden in the delamanid cohort (mean 4.41). After starting treatment, the TB burden decreased gradually in each cohort. On day 14, patients in the FDC (150 mg rifampicin, 75 mg isoniazid, 400 mg pyrazinamide, and 275 mg ethambutol hydrochloride) cohort presented a lower bacterial burden than did those in the JDB0131 cohort. However, the EBA of JDB0131 was better than that of delamanid (100 mg bid). During the time interval between days 0 and 14, JDB0131 at a dose of 200 mg bid (cohort 3) showed superior efficacy over delamanid (Fig. 2).

### Time to positivity (TTP)

**Fig. 3:**
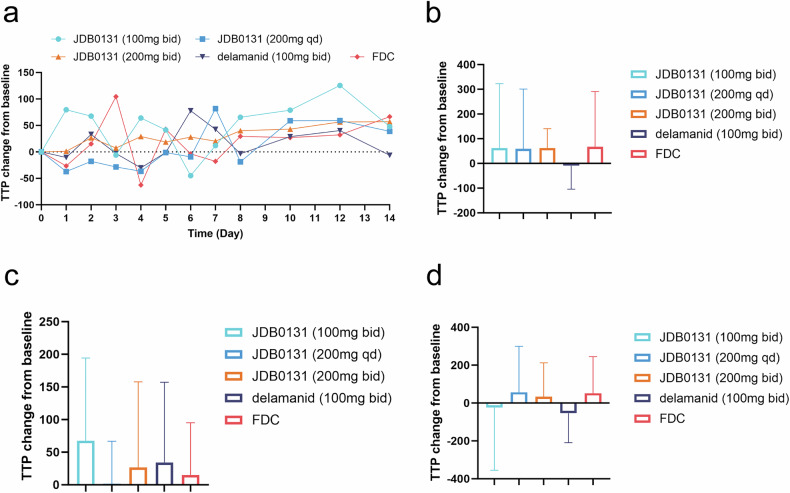
The change in time to positivity (TTP) in the liquid media culture of sputum tuberculosis patients. **a** Changes in the TTP from baseline from Day 1 to Day 14. Single data points represent the mean values of individual treatment groups. **b** The bar chart shows the changes in the TTP from baseline for each group from Day 1 to Day 14. **c** The bar chart shows the changes in the TTP from baseline for each group from Day 1 to Day 2. **d** The bar chart shows the changes in the TTP from baseline for each group from Day 2 to Day 14

The kinetics of bacterial clearance were further analyzed by measuring the TTP of sputum cultures. On day 14, data were available for 43 patients (*n* = 8 in cohort 1, *n* = 11 in cohort 2, *n* = 9 in cohort 3, *n* = 7 in the delamanid cohort, and *n* = 8 in the FDC cohort). Compared with delamanid, JDB0131 (all 3 dose levels) achieved superior TTP0--14 values. Notably, 1 patient from cohort 3 had a negative sputum culture conversion on day 15. There was variation in TTP0-2 and TTP2-14 between the JDB0131 cohort and the delamanid cohort (Fig. 3).

### Radiographic outcomes

**Fig. 4:**
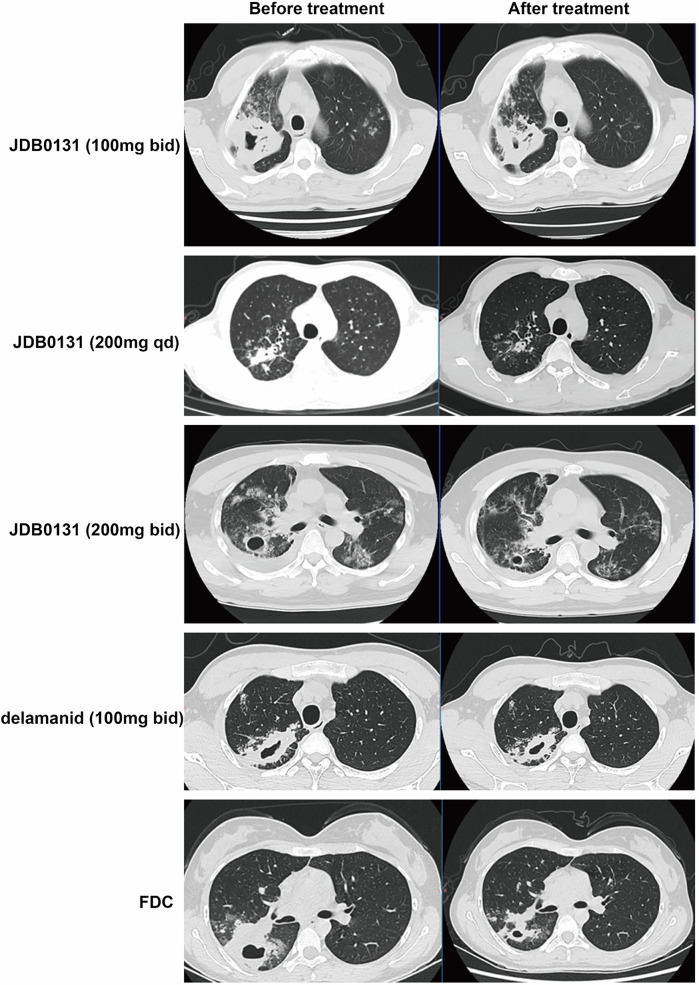
Typical radiographic images before and after treatment for each group are presented

Radiographic changes from baseline to the end of treatment were continuously evaluated via CT. Radiographic response data were available for 50 patients (*n* = 11 in cohort 1, *n* = 12 in cohort 2, *n* = 11 in cohort 3, *n* = 8 in the delamanid cohort, and *n* = 8 in the FDC cohort). In the JDB0131 cohort, 1 patient had a significantly adsorbed lesion, and another 5 had shrunk cavities (with a diameter that decreased by more than 50%, *n* = 2 in each cohort). One of the 8 patients in the FDC cohort had a decreased cavity. However, in the delamanid cohort, only 1 of 8 patients presented slightly decreased lesions (Fig. 4).

### Safety

The safety and tolerability of the treatments were evaluated by analyzing the incidence and severity of adverse events (AEs). All recruited patients (*n* = 52) were included in the safety analysis. A total of 146 adverse events were reported. Ninety-one AEs were attributed to JDB0131, involving 30 patients, without any serious AEs (SAEs). Only 3 AEs were considered drug-related (*n* = 1 in each cohort), and all were grade 1. In the delamanid cohort (*n* = 9), 30 AEs occurred (*n* = 7), including 2 SAEs (hemoptysis, *n* = 1; rash, *n* = 1). In the delamanid group, one patient with a history of hemoptysis prior to treatment experienced hemoptysis recurrence on day 6 after delamanid discontinuation, requiring emergency hospitalization. Given that hemoptysis is a well-documented complication of pulmonary tuberculosis, this event was assessed as unlikely to be related to delamanid. Another patient in the same group developed allergic dermatitis during the treatment period. Since delamanid and its metabolites are still detectable systemically at the time of onset, the event was considered potentially related to either delamanid or the concomitant standard anti-TB regimen. Another 25 AEs were reported from the FDC cohort (*n* = 8, Table 2). Among these, 5 AEs and 1 SAE (rash) were attributed to delamanid. The patient quit the study because of an SAE. The AEs observed in each cohort are listed in the supplementary materials. Notably, no QT interval prolongation was observed in any patient during treatment or follow-up.

**Table 2:** Summary safety data *across all patients*

	JDB0131 (100 mg bid) (*N* = 12)			JDB0131 (200 mg qd) (*N* = 12)			JDB0131 (200 mg bid) (*N* = 11)			delamanid (100 mg bid) (*N* = 9)			FDC (*N* = 8)			all (*N* = 52)		
Adverse events	Occurrence Instances	*n*	(%)	No.	*n*	(%)	No.	*n*	(%)	No.	*n*	(%)	No.	*n*	(%)	No.	*n*	(%)
Adverse events	25	10	83.33	36	10	83.33	30	10	90.91	30	7	77.78	25	8	100.00	146	45	86.54
Non-TEAE events	0	0	0.00	0	0	0.00	0	0	0.00	0	0	0.00	0	0	0.00	0	0	0.00
TEAE	25	10	83.33	36	10	83.33	30	10	90.91	30	7	77.78	25	8	100.00	146	45	86.54
SAE	0	0	0.00	0	0	0.00	0	0	0.00	2	2	22.22	0	0	0.00	2	2	3.85
TEAE of Grade 3 or higher	0	0	0.00	0	0	0.00	0	0	0.00	1	1	11.11	0	0	0.00	1	1	1.92
TESAE of Grade 3 or higher	0	0	0.00	0	0	0.00	0	0	0.00	1	1	11.11	0	0	0.00	1	1	1.92
TEAE related to the drug	1	1	8.33	1	1	8.33	1	1	9.09	5	4	44.44	15	7	87.50	23	14	26.92
TESAE related to the drug	0	0	0.00	0	0	0.00	0	0	0.00	1	1	11.11	0	0	0.00	1	1	1.92
TEAE leading to treatment discontinuation	0	0	0.00	0	0	0.00	0	0	0.00	0	0	0.00	0	0	0.00	0	0	0.00
TEAE leading to trial termination	0	0	0.00	0	0	0.00	0	0	0.00	0	0	0.00	0	0	0.00	0	0	0.00

Note: An event that emerges during treatment having been absent pretreatment, or worsens relative to the pretreatment state. The judgment basis for TEAEs related to the study drug is that if the relationship with the trial medication is determined as “definitely related” or “possibly related,” it is judged as a TEAE related to the study drug

*SAE* Serious adverse event, *TEAE* Treatment emergent adverse events, *TESAE* Treatment emergent serious adverse events

Data source: ADaM.ADAE

### Pharmacokinetics

Pharmacokinetic parameters were analyzed to characterize drug exposure and its relationship with dosing regimens. At 12 h post-treatment, the exposure dose of JDB0131 (AUC_0-12,ss_, ng/ml*h) in cohort 3 (7446 ± 1915) was greater than that in cohorts 1 (5165 ± 1388) and 2 (4856 ± 1513). Similarly, cohort 3 had the highest exposure (13836 ± 3970) at the time point of 24 h (AUC_0-24,ss_) compared with cohorts 1 (9408 ± 2320) and 2 (7597 ± 2687). Additionally, cohort 3 had the highest peak concentration (C_max, ss_, 658 ± 180 ng/ml*h) and longest half-life (t1/2, 13.41 ± 1.53 h). In terms of the main metabolites, cohort 3 also presented the highest exposure dose either at 12 h (AUC_0-12,ss_, 3214 ± 1004) or 24 h (AUC_0-24,ss_, 6304 ± 2034) posttreatment.

## Discussion

TB resistance remains a great challenge for current public health, and effective treatment strategies are urgently needed. Here, we report the early bactericidal effects of our novel agent JDB0131. In this phase 2a trial, 3 different doses of JDB0131 were tested for their activities and safety profiles. Compared with those of delamanid, all levels of JDB0131 presented good EBA and tolerability. Some patients achieved drastic radiographic responses. The current results warrant further phase III studies to confirm the findings in clinical practice.

Nitroimidazoles are promising antimycobacterial agents known to inhibit both aerobic and anaerobic mycobacterial activity. Under aerobic conditions, nitroimidazoles—including pretomanid—act by inhibiting mycolic acid biosynthesis, a critical process for maintaining mycobacterial cell envelope integrity, ultimately compromising structural stability and resulting in bacterial lysis.^21^ Under hypoxic or anaerobic conditions, these prodrugs undergo enzymatic reduction via mycobacterial nitroreductases (such as Ddn), generating reactive nitrogen intermediates, notably nitric oxide.^22^ This leads to respiratory inhibition and lethal oxidative stress in nonreplicating bacilli through disruption of central carbon metabolic pathways, including the pentose phosphate pathway.^23,24^ Delamanid and pretomanid are 2 nitroimidazoles approved by regulatory agencies for MDR-TB treatment.^4,5^ Pretomanid and delamanid both possess antituberculosis activity. However, both agents result in unsatisfactory absorption and QTc prolongation.^11–14^ In addition, the potential for additive QTc prolongation requires careful consideration, particularly when nitroimidazoles are used in combination with other QTc-prolonging agents, such as QT, fluoroquinolones, or clofazimine.^15,25–29^ JDB0131 is a newly developed nitroimidazole derivative with both strong activity and improved safety. JDB0131 represents a structurally optimized third-generation nitroimidazole agent that integrates the advantages of its predecessors while addressing their limitations. Notably, pretomanid or delamanid are recommended for several anti-RR/MDR-TB regimens,^30^ but in some developing countries, such as China, neither is available. Patients suffering from MDR-TB must receive combination chemotherapy, which is both lengthy and toxic, with a curation rate of only 66%.^31,32^ The emergence of JDB0131 provides a new therapeutic option for these patients, and short-course treatment regimens have become more accessible in these countries.

In our previous search for a safer nitroimidazole, we discovered JDB0131.^16^ It exhibited excellent antimycobacterial activity against *M. tuberculosis* H37Rv in vitro and in vivo, improved PK and absorption, and reduced the QT prolongation potential of delamanid. In this multicenter phase 2a trial in China, we reported the promising anti-TB activity of JDB0131, together with favorable safety profiles. In particular, it caused fewer QT-prolongation events in patients. The results were in good concordance with our preclinical data. These data support the promising future of JDB0131 as a novel anti-TB agent.

In this dose escalation trial, 3 different doses of JDB0131 were tested. Each dose was effective. Compared with each other, JDB0131 at a dose of 200 mg bid was the most effective and had acceptable toxicity. In addition, the bid dosing regimen of JDB0131 may offer an advantage in maintaining drug concentrations above the minimum inhibitory concentration (MIC) for a longer duration, thereby supporting sustained antimicrobial activity. This regimen also appears feasible for long-term clinical use. We recommend a dose of 200 mg bid for future phase III trials. However, it remains uncertain whether the prolonged half-life of JDB0131 may allow for extended dosing intervals—such as achieving a frequency of three times per week, comparable to the maintenance phase of bedaquiline regimens—which warrants further investigation.^33^

Multiple aspects can be used to evaluate antituberculosis efficacy, among which radiographic responses—particularly changes in pulmonary cavities—are considered the most indicative. The formation of the tuberculosis cavity is related to bacterial burden, virulence, and body immunity. Once formed, the tuberculosis cavity always indicates severe illness because it destroys vessel supplies to the lung, leading to a decreased drug concentration that is insufficient to inhibit bacterial growth. To overcome these changes, a combination of at least 4 effective anti-TB drugs lasting for 2 months is recommended. Five to twenty-two percent of patients have significantly smaller cavities or adsorbed lesions. A longer duration was required for complete disappearance of the cavity. However, some patients have persistent cavities. In this study, 6 patients either had lesions that had adsorbed significantly or whose cavities had decreased after JDB0131 treatment. However, only 1 patient presented with slightly decreased lesions in the delamanid cohort. These data provide supportive evidence that JDB0131 serves as a novel agent with promising rapid bactericidal activity.

Dose-dependent QT interval prolongation is a serious AE for delamanid. This was in part due to the delamanid metabolite DM6705.^13,14,34^ It is a strong hERG inhibitor and has an extremely long half-life in humans. JDB0131 generates the similar-type metabolite DM131 in animals.^16^ However, JDB0131 did not inhibit any ion channels, and DM131 had moderate inhibitory activity against hERG. In our previous study, no QT interval prolongation signal was observed in GLP toxicology studies in dogs.^16^ In vitro studies have assessed the inhibitory effects of JDB0131 and its major metabolite on the hERG potassium channel in comparison with those of delamanid and its metabolite. These results support the conclusion that JDB0131 is less likely to cause QT interval prolongation. The results of the present study provide direct clinical evidence supporting the lower cardiac toxicity of JDB0131 than delamanid.

The lower cardiac toxicity was a good explanation. Compared with delamanid, with a T1/2 of 30–38 h, JDB0131 has a much shorter T1/2. In this study, the T1/2 of JDB0131 was 11.92–18.05 h (in different cohorts). In addition, the metabolite of JDB0131 has a short T1/2. In delamanid, the T1/2 was 121–322 h.^35^ It was reasonable that the short in vivo duration of retention of JDB0131 led to fewer cardiac events.

Furthermore, the potential implications of JDB0131 in tuberculosis patients with comorbid diabetes warrant attention, as diabetes mellitus is known to exacerbate TB severity and impair treatment response.^36^ Future studies should explore the pharmacokinetics and efficacy of JDB0131 in this high-risk subpopulation to assess its utility in optimizing TB-diabetes co-management.

EBA refers to an agent’s ability to kill mycobacteria originating within pulmonary cavities during the first weeks of treatment.^10^ Usually, EBA is determined by quantifying the number of viable colony formation units (CFUs) of *M. tuberculosis* in an overnight sputum collection. Initially, this was conducted over the first 2 days of treatment, and further experience has shown that significant advantages may accrue from extending the study period from 2 to 14 days after treatment. The TTP reflects the metabolic activity of inoculated viable *M. tuberculosis* in the sputum. The TTP might represent an alternative method for estimating the activity of viable *M. tuberculosis* in sputum. The present study reported planned parallel measurements of CFU and TTP. CFU counting relies on the visual enumeration of colonies on solid media and has been the standard in EBA studies for many years. TTP is a less laborious, potentially more robust measurement using calibrated standardized equipment and fewer steps in preparing sputum for analysis. Despite the debate over which one deserves greater significance, in the current study, JDB0131 presented impressive activities in both measurements. However, owing to the influence of the limited sample size and random variation, the baseline bacterial load (CFU in sputum) differed slightly among the groups. Nevertheless, on the basis of the observed changes in EBA within each cohort, we believe that JDB0131 has promising activity, which supports further clinical development. This baseline imbalance is unlikely to have a substantial effect on the overall conclusion. Furthermore, we are planning to conduct a trial to test JDB0131 in resistant TB.

This study has several limitations. First, it comprises 5 cohorts, each containing only a limited number of patients. Second, this study enrolled only Chinese patients. These results cannot be easily extrapolated to all ethnic groups. Third, this study included 3 types of treatment regimens, namely, JDB0131, delamanid and FDC, which made comparisons of different treatments difficult. Furthermore, the twice-daily dosing regimen of JDB0131 may pose challenges to medication adherence. While this concern is important and should not be dismissed, it may be mitigated by the fact that tuberculosis patients are generally familiar with long-term pharmacotherapy. Therefore, the actual impact of the twice-daily regimen on adherence warrants formal investigation in subsequent clinical trials.

In conclusion, in this phase 2a study, we confirmed the potent anti-TB activities of our novel agent JDB0131. It has superior EBA, as shown by both TTP and CFU changes. Additionally, in the radiographic examinations, JDB0131 led to obvious alleviation of TB-related lesions. In addition, it was related to almost no toxicity related to QT interval prolongation. We recommend a dose of 200 mg bid as the suitable dose for future phase III trials. This trial identified a promising new drug for overcoming the increasing TB burden worldwide.

## Materials and methods

### Study design

This study was a multicenter, prospective randomized controlled phase 2a trial conducted to test the activity and safety of JDB0131. The anti-TB activity of the agent JDB0131 was tested in a dose escalation manner (cohort 1: 100 mg bid, cohort 2: 200 mg qd, and cohort 3: 200 mg bid). JDB0131 mesylate exhibited comparable in vivo efficacy to delamanid in preclinical studies, along with a more favorable toxicological safety profile. In the completed phase 1 multiple-dose clinical trial, JDB0131 mesylate was shown to be safe and well-tolerated. The dosing regimens in that study included 100 mg bid and 200 mg bid, which were administered once daily after meals for 14 consecutive days. Notably, steady-state plasma concentrations were achieved by day 11 in both bid dose groups, allowing day 14 pharmacokinetic data to be used for steady-state evaluations. After 14 days of administration, the plasma exposure of both bid groups to JDB0131 mesylate substantially exceeded the in vitro MIC and MBC values reported in preclinical studies. To optimize future patient adherence and dosing convenience, this phase 2a study also included a once-daily dosing arm (200 mg qd). For comparison, delamanid alone and the classic FDC regimen were included as controls. The primary endpoint was logarithmic changes in CFU in the solid media culture of sputum TB (log10 CFU). Decreasing rates of CFU (in log10 form) were used to assess the EBA. The secondary endpoint was the TTP in the liquid media culture of sputum TB. The PK of JDB0131 was set as an exploratory endpoint. The study was approved by the ethics committee of Beijing Chest Hospital (affiliated with Capital Medical School, 2023-TB04) and registered at Clinicaltrial.gov (identifier: NCT06224036).

### Patients

Patients were recruited from 3 centers in China. Enrollment was conducted via a consecutive screening process. Eligible participants were required to meet all of the following inclusion criteria: aged between 18 and 65 years; body weight ranging from 40 kg to 90 kg; a clinical diagnosis of pulmonary tuberculosis with no prior anti-TB treatment within the preceding two years and at least one positive sputum acid-fast bacilli (AFB) smear (≥1+); willingness to undergo HIV serological testing; agreement to use highly effective contraception throughout the study period (nonpregnant and nonlactating females) or appropriate contraceptive methods (males); ability to fully comprehend the trial objectives, procedures, and potential risks followed by provision of voluntary written informed consent; and demonstrated commitment to protocol compliance until study completion. The exclusion criteria included confirmed rifampin-resistant tuberculosis; seropositivity for HIV, hepatitis B surface antigen (HBsAg), hepatitis C virus (HCV) antibody, or *Treponema pallidum* antibody; presence of miliary tuberculosis or severe forms of extrapulmonary tuberculosis (e.g., meningeal, abdominal, genitourinary, or osteoarticular involvement); evidence of significant hepatic impairment, including known hepatobiliary disorders, aspartate aminotransferase (AST) or alanine aminotransferase (ALT) exceeding three times the upper limit of normal (ULN), or total bilirubin (TBIL) greater than twice the ULN; impaired renal function, indicated by a history of unstable or progressive renal disease; estimated glomerular filtration rate (eGFR) below 60 mL/min/1.73 m²; and serum creatinine levels ≥133 μmol/L (1.5 mg/dL) in men or ≥124 μmol/L (1.4 mg/dL) in women; and pregnancy or lactation.

They were randomized to receive either JDB0131, delamanid, or FDC treatment. The sample size was determined according to technique guidance from the National Medical Products Administration (NMPA) of China. Twelve patients were allocated to each JDB0131 cohort, and 8 were allocated to either the delamanid cohort or the FDC cohort. In total, 52 patients needed to be recruited. This study was open-label and without blindness. The randomization was conducted by independent statisticians via the PLAN process block randomization method (SAS V9.4 software). All patients enrolled provided written informed consent.

### Treatments

In each cohort, the agents were orally administered continuously for 14 days. The participants in the FDC group received weight-adjusted doses, whereas those in the delamanid group received a fixed dose of 100 mg bid. No dose adjustments were allowed. Sputa were collected from each patient every other day, from which TB CFU data were produced. Sputum samples were obtained through induction when necessary, as some patients ceased to produce sputum spontaneously during treatment because of varied clinical improvement. The bactericidal activity was subsequently calculated.

### Evaluation

The evaluation parameters were log_10_CFU_0-14_ (day 0–14), log_10_CFU_0-2_ (day 0–2), log_10_CFU_2-14_ (day 2–14), TTP_0-14_, TTP_0-2_, and TTP_2-14_. The log_10_CFU_X-Y_ (X, Y as different time points) was calculated as log_10_CFUX-Y =((log_10_(CFUY)−log_10_(CFUX))/(X−Y). TTPX-Y was calculated in a similar way. The PK parameters were calculated via non-compartmental analysis (NCA, WinNonlin V8.4). A power model was used to analyze the linear correlation between the dose and the AUC or C_max_.

### Statistics

Continuous variables are expressed as median values with ranges or as the means ± standard deviations, depending on the data distribution. Categorical variables are summarized as proportions. The data were analyzed via power models and one-way ANOVA followed by post hoc comparisons where appropriate. Statistical analyses were performed via SAS (version 9.4).

## Supplementary information

SUPPLEMENTAL MATERIAL OF JDB0131

## Data Availability

To protect patient privacy, CT scans and pathological images are not publicly available. However, deidentified participant data (excluding imaging data), the study protocol, and the statistical analysis plan can be made available upon reasonable request within 2 years following the publication of this paper. Qualified researchers seeking access to individual patient-level clinical data may submit requests to the corresponding author. Additional supporting data are accessible in the article and Supplementary Information.
