# Supplementary material for: A novel antituberculosis agent exhibits potent clinical efficacy and good safety profile: an open-label, randomized-controlled, multicenter, phase 2a trial
Source: Signal Transduct Target Ther. 2025 Dec 30;10:427. doi: 10.1038/s41392-025-02517-z (PMC12748780; doi:10.1038/s41392-025-02517-z)
Supplement: Supplementary file 1 — SUPPLEMENTAL MATERIAL OF JDB0131 [file 41392_2025_2517_MOESM1_ESM.docx]

Supplementary Materials for

JBD0131, a novel anti-tuberculosis agent, exhibits potent clinical efficacy and good safety profile: an open-label, randomize-controlled, multicenter, phase 2 trial

Chu Naihui1*#, Nie Wenjuan1#, Du Juan5#, Wang Manni2, Ma Liping1, Wang Qingfeng1, Wang Jun1, Hu Xiaomeng5, Jin Wu5, Lu Yu1, Huang Mailing1, He Jianqing3*, Ding Zhenyu2*, Wei Xiawei4*

Correspondence to: [chunaihui1994@sina.com](mailto:chunaihui1994@sina.com); [jianqing_he@scu.edu.cn](mailto:jianqing_he@scu.edu.cn); [dingzhenyu@scu.edu.cn](mailto:dingzhenyu@scu.edu.cn); xiaweiwei@scu.edu.cn

**This PDF file includes:**

Materials and Methods

CONSORT checklist

Methods

**Protocol Summary**

| **Protocol Number** | JDB-131-201 |
| --- | --- |
| **Version/Date** | V4.0 / May 15, 2024 |
| **Sponsor** | Chengdu JDB PharmaSilver Medical Technology Co., Ltd. |
| **Clinical Research Sites** | - Beijing Chest Hospital, Capital Medical University - West China Hospital, Sichuan University - Wuhan Pulmonary Hospital (Wuhan Institute for Tuberculosis Control) |
| **Study Type** | Phase IIa Clinical Study |
| **Clinical Trial Approval No** | CXHL1700246; CXHL1700247 (Clinical Trial Notification No.) |
| **Investigational Drug** | - Name: JDB0131 Besylate - Dosage Form: Tablet - Strength: 50 mg |
| **Indication** | Drug-resistant tuberculosis (TB) |
| **Study Title** | A Randomized, Open-label, Multicenter, Phase IIa Clinical Study to Evaluate the Early Bactericidal Activity, Safety, Tolerability, and Pharmacokinetic Characteristics of JDB0131 Besylate Tablets in Patients with Drug-Susceptible Pulmonary Tuberculosis |
| **Study Objectives** | **Primary Objective:**  To evaluate the early bactericidal activity (EBA) of JDB0131 besylate tablets in patients with drug-susceptible pulmonary TB.  To explore the optimal effective dose to inform the design of Phase IIb clinical trials.  **Secondary Objectives:**  To assess the safety and tolerability of JDB0131 besylate tablets following multiple-dose administration in patients with drug-susceptible TB.  To characterize the pharmacokinetic (PK) profile of JDB0131 besylate tablets after multiple-dose administration in this patient population. |
| **Study Design** | This is a randomized, open-label, active-controlled study to evaluate the early bactericidal activity (EBA), safety, tolerability, and pharmacokinetics (PK) of JDB0131 besylate tablets in patients with drug-susceptible pulmonary tuberculosis (TB).  **Study Groups (5 Arms)：**  JDB0131 besylate 100 mg BID (twice daily)  JDB0131 besylate 200 mg QD (once daily)  JDB0131 besylate 200 mg BID (twice daily)  Fixed-dose combination (FDC) anti-TB drugs (dose adjusted by body weight, see Table 1)  Delamanid 100 mg BID (active comparator)  **Table 1: FDC Anti-TB Drug Dosing Based on Body Weight**   \| Body weight（kg） \| Number of Tablets (QD) \| \| --- \| --- \| \| 30 ~ 37 \| 2 \| \| 38 ~ 54 \| 3 \| \| 55 ~ 70 \| 4 \| \| 71 and above \| 5 \|   FDC Composition per Tablet: Rifampin (R) 150 mg, Isoniazid (H) 75 mg, Pyrazinamide (Z) 400 mg, Ethambutol (E) 275 mg  **Sample Size:**  JDB0131 dose groups: 12 participants each  FDC anti-TB drug group and delamanid group: 8 participants each  Total planned enrollment: 52 participants (male and female)  Participants will be randomly assigned to one of the 5 groups. They will be hospitalized at the clinical trial center by 4:00 PM on Day -2 (D-2) and undergo 14 consecutive days (D1–D14) of multiple-dose administration:   - For BID groups: Twice-daily dosing (D1–D14) - For QD groups: Once-daily dosing (D1–D14)   On Day 15 (D15), after completing PK sample collection and safety assessments, participants may be discharged (or the next day if evaluations are completed late). They will then be referred for standard anti-TB treatment. If safety assessments reveal abnormalities, the investigator will determine clinical significance and provide appropriate management.  Follow-up visits will occur on:   - Day 18 (D18) - Day 19 (D19) (JDB0131 groups only) - Day 21 (D21) - Day 28 (D28±1)   During these visits, PK samples will be collected, and safety will be assessed. Abnormal findings will be evaluated by the investigator for clinical relevance, with follow-up actions as needed. A telephone follow-up will be conducted on Day 35±3 after the last dose to further assess safety and tolerability.  **Randomization**: Participant randomization numbers will be generated by an independent statistician using the PLAN procedure in SAS V9.4 (block randomization method). |

| **Dosing Regimen** | - BID Groups (D1–D14):   Dosing twice daily, with 12±2 hours between doses (strict 12±0.5 hours on D1 and D14 for JDB0131 groups).Participants must consume a standardized meal 30 minutes before dosing, followed by the study drug with ~240 mL warm water.   - QD Groups (D1–D14):   Once-daily dosing in the morning.Participants must consume a standardized meal 30 minutes before dosing, followed by the study drug with ~240 mL warm water.Exception: FDC anti-TB drug group participants must take medication 1 hour ±15 minutes before breakfast.  Water Restriction (JDB0131 Groups):  No water intake 1 hour before to 1 hour after dosing (except for drug administration). Clinical trial sites unable to provide standardized meals may allow participants to self-prepare meals. |
| --- | --- |
| **Study Duration** | 12 months. |
| **Study Population / Sample Size** | Planned enrollment: 52 adult participants with drug-susceptible pulmonary TB (male and female). |
| **Inclusion Criteria (All must be met)** | 1. **Age**: 18–65 years (no gender restriction). 2. **Weight**: 40–90 kg. 3. **Diagnosis**: Clinically confirmed pulmonary TB, with no anti-TB treatment in the past 2 years, and at least one positive sputum AFB smear (≥1+). 4. **HIV Testing**: Willing to provide a blood sample for HIV testing. 5. **Contraception**:    - Non-pregnant, non-lactating females must agree to use highly effective contraception throughout the study.    - Males must agree to use appropriate contraceptive methods during the study. 6. **Informed Consent**: Participants must fully understand the study’s purpose, procedures, and potential risks, and voluntarily sign the informed consent form. 7. **Compliance**: Willing to complete the study per protocol requirements. |

| **Exclusion Criteria (Any single criterion excludes participation)** | 1. **Rifampin resistance**. 2. **Positive for**:    - HIV antibody    - Hepatitis B surface antigen (HBsAg)    - Hepatitis C virus (HCV) antibody    - Syphilis (Treponema pallidum antibody) 3. **Severe TB forms**:    - Miliary TB (investigator-determined)    - Extrapulmonary TB (e.g., TB meningitis, abdominal TB, genitourinary TB, bone/joint TB) 4. **Hepatic impairment:**    - Chronic active hepatitis, severe liver dysfunction    - AST or ALT >3× ULN    - Total bilirubin (TBIL) >2× ULN 5. **Renal impairment**:    - Unstable/progressive renal disease    - Moderate/severe renal impairment (eGFR <60 mL/min/1.73m²)    - Serum creatinine:      1. Males: ≥133 μmol/L (1.5 mg/dL)      2. Females: ≥124 μmol/L (1.4 mg/dL) 6. **QT prolongation risk**:    - Family history of long QT syndrome    - Current use of QT-prolonging drugs (e.g., quinidine, procainamide, amiodarone, sotalol) 7. **ECG abnormalities**:    - QTcF >450 ms (Fridericia correction)    - Pathological Q waves (>40 ms or depth >0.4–0.5 mV)    - Wolff-Parkinson-White syndrome    - Left/right bundle branch block    - 2nd-/3rd-degree heart block    - Intraventricular conduction delay (QRS >120 ms)    - Sinus bradycardia (<50 bpm) 8. **Cardiovascular history (past 6 months)**:    - Myocardial infarction    - Cardiac surgery or coronary revascularization (CABG/PTCA)    - Unstable angina    - Congestive heart failure (NYHA Class III/IV)    - Transient ischemic attack or severe cerebrovascular disease 9. **Dietary restrictions**: Inability to comply with standardized meals due to allergies/special needs. 10. **GI surgery**: History of procedures affecting drug absorption/excretion. 11. **Ophthalmologic findings**: Clinically significant abnormalities (investigator-determined). 12. **Depression**: Hamilton Depression Scale (17-item) score >7. 13. **Unstable medical conditions**: Severe cardiovascular, renal, hepatic, hematologic, oncologic, endocrine, psychiatric, or rheumatic diseases (investigator-determined). 14. **Substance restrictions**: Inability to abstain from alcohol, caffeine/xanthine-containing products (e.g., coffee, tea, cola, chocolate) from **48h pre-dose until final PK sampling**. 15. **Sputum volume**: Expected overnight sputum volume <8 mL. 16. **Recent anti-TB drugs**: Use of drugs with anti-mycobacterial activity (e.g., levofloxacin, moxifloxacin, clarithromycin, amikacin) within **5 half-lives** prior to dosing. 17. **Recent investigational drugs**: Use within **3 months** prior to dosing. 18. **Substance abuse**: Alcohol/drug dependence within **6 months** (investigator-assessed risk). 19. **Psychotropic drugs**: Use of barbiturates, opioids, or phenothiazines within **30 days**. 20. **Chronic steroids**: Systemic corticosteroid use (>4 weeks cumulative) within **3 months** prior to enrollment. 21. **Allergies**: Hypersensitivity to study drugs or related compounds. 22. **CYP450 modulators**: Strong inducers/inhibitors (e.g., carbamazepine, phenytoin, rifampin, clarithromycin, ritonavir, ketoconazole) within **30 days** pre-treatment. 23. **Pregnancy/lactation**: Positive pregnancy test or breastfeeding. 24. **Live vaccines**: Received within **4 weeks** prior to dosing (inactivated vaccines permitted). 25. **Investigator discretion**: Any condition affecting compliance, data interpretation, or participant safety. |
| --- | --- |

| **Withdrawal Criteria** | Participants may voluntarily withdraw at any time. Investigators may also withdraw participants for:   1. Withdrawal of consent. 2. Protocol violations. 3. Adverse events (AEs) necessitating discontinuation. 4. Poor compliance affecting data integrity. 5. Loss to follow-up. 6. Ethical/medical concerns (investigator-determined). |
| --- | --- |
| **Study Termination Criteria** | The trial or site may be terminated for:   1. Major protocol/GCP violations compromising data integrity. 2. Sponsor decision (e.g., administrative/funding reasons). 3. Regulatory/ethics committee mandate. |
| **Primary Endpoints** | - Change in log_10_ colony-forming units (log_10_ CFU) of *Mycobacterium tuberculosis* in solid culture (0–14 days) - Change in log_10_ CFU of *M. tuberculosis* in solid culture (0–2 days) - Change in log_10_ CFU of *M. tuberculosis* in solid culture (2–14 days) |
| **Secondary Endpoints** | **Safety and Tolerability Assessments:**  • Incidence and severity of adverse events (AEs)  • Incidence of serious adverse events (SAEs) and unexpected SAEs  • Vital signs  • Physical examinations  • 12-lead electrocardiogram (ECG)  • Laboratory tests  • Ophthalmologic examinations and depression scale scores  **Pharmacokinetic (PK) Parameters:**  • Time to maximum concentration (T _max_ )  • Maximum concentration (C _max_ )  • Area under the plasma concentration-time curve from 0 to 12 hours (AUC _0–12_ )  • Time to steady-state maximum concentration (T _ss,max_ )  • Steady-state maximum concentration (C _ss_,max )  • Steady-state trough concentration (C _ss,min_ )  • Average steady-state concentration (C _ss,avg_ )  • Elimination half-life at steady-state (t _1/2,ss_ )  • AUC from 0 to 12 hours after the last dose (AUC _0–12,ss_ )  • AUC from 0 to the last measurable time point after the last dose (AUC _0–12,ss_ )  • AUC extrapolated to infinity after the last dose (AUC _0–∞,ss_ )  • Apparent volume of distribution at steady-state (V _d,ss_ )  • Oral clearance at steady-state (CL _ss_ )  • Change in log_10_ colony-forming units (log_10_ CFU) of Mycobacterium tuberculosis in solid culture (0–14 days)  • Change in log_10_ CFU of M. tuberculosis in solid culture (0–2 days)  • Change in log_10_ CFU of M. tuberculosis in solid culture (2–14 days)  • Accumulation ratio:  • R _ac_ (C _max_ ) = C _max,ss_ (Day 14) / C _max_ (Day 1)  • R _ac_ (AUC) = AUC _0–12,ss_ (Day 14) / AUC _0–12_  (Day 1)  • Fluctuation index: % fluctuation at steady-state = 100 × (C _ss,max_ − C _ss,min_ ) / C _ss,avg_  **Efficacy Assessments:**   - Change in time to positivity (TTP) of *M. tuberculosis* in liquid culture (0–14 days) - Change in TTP in liquid culture (0–2 days) - Change in TTP in liquid culture (2–14 days) - Time-course change in log10 CFU of *M. tuberculosis* in solid culture (0–14 days)   Time-course change in TTP of *M. tuberculosis* in liquid culture (0–14 days) |
| **Biological Sample Collection** | Procedures for collection, processing, transport, and storage follow the study’s standard operating procedures (SOPs).  **Sputum Sample Collection:**   - Pre-treatment: Overnight sputum (6:00 PM–8:00 AM) collected on D-2 and D-1. - Post-treatment: Overnight sputum collected on Days 1, 2, 3, 4, 5, 6, 7, 8, 10, 12, and 14 (13 total collections). - Handling:   - Sputum is collected in pre-labeled, wide-mouth, screw-cap containers and stored at 2–8°C.   - After collection, samples are transported on ice to the lab for:     1. Volume measurement and aliquoting.     2. TTP testing (liquid culture).   - Remaining aliquots are stored at 2–8°C and shipped to the central lab within 72 hours for CFU counting.   **PK Blood Sampling (*Not performed for FDC anti-TB or delamanid groups*):**   - QD Groups:   - Day 1 (D1): Pre-dose (0 h), 2 h ± 3 min, 4 h ± 3 min, 8 h ± 15 min, 12 h ± 15 min, 24 h ± 1 h (D2).   - Days 6, 8, 10: 120 h ± 1 h (D6), 168 h ± 1 h (D8), 216 h ± 1 h (D10).   - Day 14 (D14): Pre-dose (0 h), 2 h ± 3 min, 4 h ± 3 min, 8 h ± 15 min, 12 h ± 15 min, 24 h ± 1 h (D15).   - Follow-up: 96 h ± 1 h (D18), 120 h ± 1 h (D19), 168 h ± 1 h (D21). - BID Groups: Additional sampling at 16 h ± 0.5 h (4 h ± 0.5 h post-second dose) on D1 and D14. - Procedure:   - Collected via venipuncture or indwelling catheter (discard 1 mL initially).   - 3 mL whole blood per sample in lithium heparin tubes, processed per SOP for PK analysis.   **PK Urine Sampling (*Not performed for FDC anti-TB or delamanid groups*):**   - Pre-dose: Single void within 6 h before first dose. - Post-dose: 0–12 h, 12–24 h, 24–36 h, and 36–48 h collections. - Purpose: Quantify JDB0131 besylate and metabolites to characterize urinary excretion profiles. |

| **Statistical Analysis** | **General Analysis**  Unless otherwise specified, statistical analyses will be performed using SAS V9.4 or higher or WinNonlin V7.0 or higher.   1. Continuous variables: Descriptive statistics including:    - Number of observations (n)    - Arithmetic mean (Mean)    - Standard deviation (SD)    - Median (Median)    - Minimum (Min) and maximum (Max)    - For PK concentration and parameter data:      1. Coefficient of variation (%CV)      2. Geometric mean (GeoMean)      3. Lower and upper quartiles (Q1, Q3)      4. Log-standard deviation (SD log) or geometric coefficient of variation (%CVb) 2. **Categorical variables**:    - Frequency counts (**n**) and percentages (**%**) 3. **Data presentation**:    - Appropriate **statistical tables and figures** summarizing efficacy, safety, and PK data.    - **Baseline characteristics** of enrolled participants will be summarized descriptively by dose group.    - **Participant disposition** (enrollment, discontinuations) will be reported.   **Analysis Sets**  Full Analysis Set (FAS)   - - Includes all randomized participants.   - Used for demographics, baseline characteristics, and discontinuation analysis.   - Summarized by randomized treatment group.   Intent-to-Treat Set (ITTS)   - - Includes all randomized participants who received the study drug and had post-dose efficacy data.   - Used for efficacy analysis.   - Summarized by actual treatment and dose received.   Per-Protocol Set (PPS)   - - Includes randomized participants who received the study drug, had post-dose efficacy data, and no major protocol deviations.   - Used for confirmatory efficacy analysis.   - Summarized by actual treatment and dose received.   Safety Analysis Set (SAS)   - - Includes all randomized participants who received the study drug and had post-dose safety data.   - Used for safety and tolerability analysis.   - Summarized by actual treatment group.   **PK Concentration Set (PKCS)**   - - Includes randomized participants who received **JDB0131 besylate** and had **at least one PK concentration measurement**.   - Summarized by **actual treatment group**.   **PK Parameter Set (PKPS)**   - - Includes randomized participants who:     1. Received **JDB0131 besylate**.     2. Had **no major protocol violations affecting PK evaluation (e.g., C** **max** **, AUC)**.     3. Had **at least one evaluable PK parameter**.   - Summarized by **actual treatment group**.   **Final determination of analysis sets** will be made during the **database lock meeting**.  **Efficacy Evaluation**   - CFU and TTP calculations:   - For each time point, the mean of up to 4 CFU counts and mean of up to 2 TTP values will be calculated.   - Early Bactericidal Activity (EBA):     - Calculated for CFU and TTP using:   EBA(CFU)_day_ _y_ _–_ _x_ = （(log_10_(CFU_day_ _y_)-log_10_(CFU_day_ _x_))/(x-y)   - - - Similar formula applies for TTP-based EBA. - Statistical comparisons:   - One-way ANOVA will be used for exploratory comparisons of EBA across JDB0131 dose groups.   - No formal statistical testing will be performed between JDB0131, FDC anti-TB, and delamanid groups.   **Pharmacokinetic (PK) Evaluation**   - Concentration-time profiles:   - Individual and mean plasma concentration-time curves (linear and semi-log scales) will be plotted.   - Actual sampling times will be used for individual plots; planned sampling times for mean plots. - Non-compartmental analysis (NCA):   - Conducted using WinNonlin V7.0 or higher.   - PK parameters for JDB0131 besylate and major metabolites will be calculated. - Dose proportionality:   - Power model will assess linearity between dose and PK parameters (C max , AUC)**.**   **Safety and Tolerability Evaluation**   - Primary approach: Descriptive statistics (SAS). - Reporting includes:   - Adverse events (AEs) and adverse drug reactions (ADRs) by dose group and time period.   - Physical examinations, vital signs, ECG, laboratory tests, ophthalmologic exams, and depression scale scores (changes from baseline). - Special focus:   - Clinically significant abnormalities.   - Shifts from baseline in safety parameters. |
| --- | --- |

CONSORT checklist

CONSORT 2025 expanded checklist of detailed information to include when reporting a randomised trial

| **Section / Topic** | **No** | **CONSORT 2025 checklist item**  **description** | **Detailed item description** | **Reported on Page No** |
| --- | --- | --- | --- | --- |
| **Title and abstract** |  |  |  |  |
| Title and structured abstract | 1a | Identification as a randomised trial | Use the word “randomised” in the title | Page 1 |
|  | 1b | Structured summary of the trial design, methods, results, and conclusions | - Specific objectives - Trial design (e.g., parallel group, cluster) and framework (e.g., superiority, equivalence, non- inferiority, exploratory) - Methods:   - Eligibility criteria for participants and settings where the trial was conducted   - Intervention(s) and comparator(s) intended for each group   - Primary outcome(s)   - How participants were allocated to interventions (e.g., centralised computer- generated randomisation)   - Who was blinded after assignment to interventions (e.g., participants, care providers, outcome assessors) - Results:   - Number of participants randomised to each group   - For the primary outcome, the number of participants analysed in each group   - For the primary outcome, a result for each group and the estimated effect size and its precision   - Important harms or unintended events for each group - Conclusions:   - General interpretation of the results - Name of trial register and identification number - Sources of funding   *Do not report information that does not appear in the body of the paper* | Page 3 |
| **Open science** |  |  |  |  |
| Trial registration | 2 | Name of trial registry, identifying number (with URL) and date of  registration | - Name of registry - Trial registry identifying number - URL to registry record | Page 15 |

|  |  |  | - Date of registration - Whether the trial results are already publicly posted to the trial registry, as a preprint (with URL citation) or published articles (with citations) |  |
| --- | --- | --- | --- | --- |
| Protocol and statistical analysis plan | 3 | Where the trial protocol and statistical analysis plan can be accessed | - Where the protocol can be accessed with URL to its location (e.g., publication with DOI, repository such as Open Science Framework (OSF), trial registry, supplement to the trial report) - Where the full statistical analysis plan can be accessed with URL to its location (e.g., publication with DOI, repository such as Open Science Framework, trial registry,   supplement to the trial report) | Supplementary materials |
| Data sharing | 4 | Where and how the individual de- identified participant data (including data dictionary), statistical code and any other materials can be accessed | - What data and materials are shared, for example:   - De-identified participant data, data dictionary, analytical code used to process the data   - Materials associated with the intervention (e.g., handbook or video for non- pharmacological interventions) - Where the data and materials are accessible (e.g., upon request, through a data sharing platform) - How the data and materials are shared (e.g., application process to access the data) - If no sharing is planned, this should be clearly stated with an explanation | Page 18 |
| Funding and conflicts of interest | 5a | Sources of funding and other support (e.g., supply of drugs), and role of funders in the design, conduct, analysis and reporting of the trial | - Name of funder(s) - Type of funding:   - Direct monetary support   - Indirect support (free trial drugs, equipment, or services such as statistical analysis or use of medical writers) - Role of the funder(s) in the trial design, conduct, data analysis and reporting | Page 19 |
|  | 5b | Financial and other conflicts of interest of the manuscript authors | - Conflicts of interests of the trial manuscript authors, including:   - Financial: salary support or grants; ownership of stock or options; honoraria (e.g., for advice, authorship, or public speaking); paid consultancy or service on advisory boards; and holders of patents or patents pending   - Non-financial: academic commitments; personal or professional relationships; other affiliations with special interests or advocacy positions - Any procedures to reduce the influence of conflicts of interest on the trial’s design,   conduct, analysis, or reporting | Page 19 |

|  |  |  | - If no conflicts of interest, this should be clearly stated |  |
| --- | --- | --- | --- | --- |
| **Introduction** |  |  |  |  |
| Background and rationale | 6 | Scientific background and rationale | - Importance of the research question   - Why a new trial is needed in the context of available evidence     - Explanation of how the intervention might work     - Justification of the choice of comparator     - Evidence of the benefits and harms of the intervention     - Reference to systematic review(s) of relevant trials where available | Page 4 |
| Objectives | 7 | Specific objectives related to benefits and harms | - Trial objective(s) related to benefits and harms including:   - Participants   - Intervention(s)   - Comparator(s)   - Primary outcome(s)   - Timepoint of primary outcome - If the trial was designed using the estimands framework, the objectives should be   defined in terms of this framework | Page 4 |
| **Methods** |  |  |  |  |
| Patient and public involvement | 8 | Details of patient or public involvement in the design, conduct and reporting of the trial | - How patients and the public were involved at different trial stages (e.g., design, conduct, reporting) - Who was involved (e.g., patients, carers, or members of the public) - If no patient or public involvement, this should be stated | Page 16 |
| Trial design | 9 | Description of trial design including type of trial (e.g., parallel group, crossover), allocation ratio, and framework (e.g., superiority,  equivalence, non-inferiority, exploratory) | - Type of trial design (e.g., parallel group) - Conceptual framework (e.g., superiority, non-inferiority, or equivalence) - Unit of randomisation (e.g., individual participant) - Allocation ratio (e.g., 1:1) | Page 14 |
| Changes to trial protocol | 10 | Important changes to the trial after it commenced including any outcomes or analyses that were not prespecified, with reason | - Any changes to the original protocol after the trial commenced with timing and reasons, e.g., in randomisation ratio, eligibility criteria, interventions, outcomes (method of assessment, timepoint, changes of outcomes), target sample size, number of trial groups, duration of follow-up, analysis methods, trial conduct (such as dropping a site with poor data quality), any other important changes - Any outcomes that were not prespecified - Any analyses that were not prespecified | N/A |

| Trial setting | 11 | Settings (e.g., community, hospital) and locations (e.g., countries, sites) where the trial was conducted | - Location(s) where the trial was carried out (e.g., country, city) - Setting of participant recruitment (e.g., primary or tertiary care; outpatient community or hospital clinic, inpatient unit) - Number of sites | Page 14 |
| --- | --- | --- | --- | --- |
| Eligibility criteria | 12a | Eligibility criteria for participants | - All inclusion and exclusion criteria - Methods of recruitment (e.g., referral or self-selection; advertisements) | Page 16 |
|  | 12b | If applicable, eligibility criteria for sites and for individuals delivering the interventions (e.g., surgeons, physiotherapists) | If applicable:   - Eligibility criteria for sites, for example the sites volume for the procedure - Eligibility criteria for individuals delivering the interventions (e.g., surgeons, physiotherapists), such as professional qualifications, years in practice, skills, validation of specific training before trial initiation | N/A |
| Intervention and comparator | 13 | Intervention and comparator with sufficient details to allow replication. If relevant, where additional materials describing the intervention and comparator (e.g., intervention manual) can be accessed | - Details of each intervention and comparator to allow replication including for example:   - Components of the intervention and comparator   - How they were administered   - When and for how long they were administered   - Any procedure for tailoring the intervention/comparator to individual participants   - Any physical or informational materials used as part of the intervention/comparator (e.g., instruction manual) and where the materials can be accessed   - Where appropriate, relevant concomitant care and interventions that are allowed (e.g., rescue interventions) or prohibited during the trial   - Where appropriate, criteria used to guide modifications to the trial intervention/comparator (e.g., drug dose change in response to harms, participant request, or improving/worsening disease) and discontinuations of the trial intervention/comparator - When the comparator group is “usual care”:   - Description of usual care   - Whether the intervention group(s) also received usual care - When and how fidelity of care providers and adherence of participants to the intervention/comparator protocols were assessed, if applicable - Any strategies for improving fidelity of care providers and adherence of participants to the intervention/comparator protocols | Page 17 |

|  |  |  | - Where appropriate, prespecified definition for classifying participants as being treated   as planned or not |  |
| --- | --- | --- | --- | --- |
| Outcomes | 14 | Pre-specified primary and secondary outcomes, including the specific measurement variable (e.g., systolic blood pressure), analysis metric (e.g., change from baseline, final value, time to event), method of aggregation (e.g., median, proportion), and time point for each outcome | - Which outcomes are primary and secondary as prespecified in the protocol - Rationale for the choice of trial outcomes and whether they are part of a core outcome set - For each outcome:   - Specific variable measured (e.g., Beck Depression Inventory score) with definition where relevant (e.g., major bleeding was defined as fatal bleeding or symptomatic bleeding in a critical area or organ; all-cause mortality as per hospital database)   - Analysis metric for each participant (e.g., change from baseline, end value, time-to- event)   - Method of aggregation for each trial group (e.g., median, proportion with score > 2)   - Timepoint of interest for analysis (e.g., 3 months)   - Who assessed outcomes (patient, doctor, nurse, caregiver, other) | Supplementary materials |
| Harms | 15 | How harms were defined and assessed (e.g., systematically, non- systematically) | - For each systematically assessed harm (active/targeted surveillance):   - Definition and measurement (e.g., name of a validated questionnaire)   - Where appropriate, the metrics, method of aggregation and time point of interest for analysis (see item 14)   - Procedures for harms assessment, including:     - Who did the assessment, and whether they were blinded to the allocated trial group     - Assessment time points and the overall time period for recording harms - For each non-systematically assessed harm (passive surveillance):   - How data were collected   - Assessment time points and overall time period for recording harms   - Process for coding each harm and grading its severity, including:     - Who did the coding and severity grading, and whether they were blinded to the allocated trial group     - Which coding and severity grading systems were used, if any - For grouping of harms by seriousness, severity, body system, withdrawals (due to harms), and causality:   - Definitions of grouping categories | Supplementary material |

|  |  |  | - Who did the grouping, and whether they were blinded to the allocated trial   group |  |
| --- | --- | --- | --- | --- |
| Sample size | 16a | How sample size was determined, including all assumptions supporting the sample size calculation | - Primary outcome on which the calculations are based - Outcome values (e.g., proportion) assumed for each group, with rationale or supporting references - Target difference in outcome values between trial groups (including common standard deviation for continuous outcomes), with rationale - Statistical significance level or α (type I) error - Statistical power or β (type II) error - Any adjustments to account for e.g., missing data or non-adherence - Target sample size per trial group - Any software used for sample size calculation | Supplementary material |
|  | 16b | Explanation of any interim analyses and stopping guidelines | Interim analyses:   - Whether interim analyses were conducted - Whether interim analyses were pre-planned - When they were conducted (timing and indications), and by whom - Statistical methods - Who had access to interim results, and whether they were blinded - Whether an independent Data Monitoring Committee was involved Stopping guidelines: - Any criteria (statistical or non-statistical) used to inform decisions about early stopping or other adaptations (e.g., sample size re-estimation) - Who made the decision to continue, stop, or modify the trial | Supplementary material |
| Randomisation: |  |  |  |  |
| Sequence generation | 17a | Who generated the random allocation sequence and the method used | - Who generated the allocation sequence - Method of sequence generation (e.g., computerised random number generator) - Any software used for random sequence generation | Supplementary material |
|  | 17b | Type of randomisation and details of any restriction (e.g., stratification, blocking and block size) | - Type of randomisation: simple versus restricted (e.g., blocked); fixed versus adaptive (e.g., minimisation, urn); and where relevant, the reasons for such choices - Methods used for restriction:   - Block randomisation:     - How blocks were generated (e.g., permuted block design with a   computer random number generator) | Supplementary material |

|  |  |  | - Block size(s) - Whether block size(s) was fixed or randomly varied - Whether trialists were or became aware of block size(s) - Stratification:   - Factors of stratification (e.g., site, sex, disease stage) - Minimisation:   - Factors incorporated   - Whether a random element was used, and details |  |
| --- | --- | --- | --- | --- |
| Allocation concealment mechanism | 18 | Mechanism used to implement the random allocation sequence (e.g., central computer/telephone; sequentially numbered, opaque, sealed containers), describing any steps to conceal the sequence until  interventions were assigned | - How the individuals enrolling participants were made unaware of the next trial group assignment in the random sequence (not to be confused with blinding) | Supplementary material |
| Implementation | 19 | Whether the personnel who enrolled and those who assigned participants to the interventions had access to the random allocation sequence | - Who had access to the random allocation sequence - Who enrolled participants - Who assigned participants to interventions - Whether personnel enrolling and assigning participants had access to the random allocation sequence. - When individuals involved in sequence generation and allocation concealment are the same individuals involved in the implementation of assignment:   - How and where the allocation list was stored   - Any mechanisms to prevent those enrolling and assigning participants from   accessing the list (e.g., allocation sequence was locked in a secure location) | Supplementary material |
| Blinding | 20a | Who was blinded after assignment to interventions (e.g., participants, care providers, outcome assessors, data analysts) | Who was blinded to trial group assignments:   - Trial participants - Care providers (i.e., those administering the intervention) - Data collectors (those collecting data on the trial outcomes) - Outcome assessors (i.e., those who determine if a participant experienced the outcome of interest), e.g., the participant for patient reported outcomes, the care provider, an independent researcher - Data analysts performing the statistical analysis | Supplementary material |

|  | 20b | If blinded, how blinding was achieved and description of the similarity of interventions | - Mechanism used to establish blinding (e.g., identical placebo, double-dummy) - Any similarities or differences of the characteristics (e.g., appearance, taste) of the interventions being compared - Any procedures intended to maintain blinding and reduce risk of unblinding, when appropriate - Any procedures intended to evaluate blinding procedures (e.g., pre-trial testing of blinding procedures) - Any known compromises in blinding (e.g., unblinding of any participants or caregivers during the trial) - If done, any emergency unblinding with reasons and the procedure used | Supplementary material |
| --- | --- | --- | --- | --- |
| Statistical methods | 21a | Statistical methods used to compare groups for primary and secondary outcomes, including harms | - Statistical methods for each analysis:   - Main analysis methods for statistical comparison   - Any deviation from the statistical analysis plan   - Distinction between prespecified and post-hoc analyses   - Effect measure (e.g., absolute risk) with confidence intervals   - Statistical significance level - For Bayesian analysis: choices of priors, computational choices, details of any modelling, effect measure with credible intervals - For adjusted analyses (if applicable):   - Rationale for adjusted analyses   - Whether adjusted analyses were pre-specified or post hoc   - Choice of covariates adjusted for   - Statistical methods (including how continuous covariates were handled) - Methods to account for multiplicity, if applicable - Software used for analyses | Page 17  Supplementary material |
|  | 21b | Definition of who is included in each analysis (e.g., all randomised participants), and in which group | - Who was included in the primary and other analyses (e.g., all randomised participants with either observed or imputed outcome data):   - Any exclusions due to missing data or other reasons - Trial group in which participants were analysed (e.g. , as-randomised) | Supplementary material |

|  | 21c | How missing data were handled in the analysis | For each analysis:   - Assumption about the missing data mechanism (e.g., missing at random) with justification - How missing data were handled (e.g., multiple imputation, model-based approaches), with justification - Whether sensitivity analyses were conducted | Supplementary material |
| --- | --- | --- | --- | --- |
|  | 21d | Methods for any additional analyses (e.g., subgroup and sensitivity analyses), distinguishing prespecified from post-hoc | - Whether additional analyses were pre-specified or post hoc (i.e., suggested by the data) - Whether all additional analyses conducted are reported For sensitivity analyses: - Rationale - Statistical methods For subgroup analyses: - Baseline variables explored - Rationale - Statistical methods (e.g., test of interaction) | N/A |
| **Results** | | |  |  |
| Participant flow, including flow diagram | 22a | For each group, the numbers of participants who were randomly assigned, received intended intervention, and were analysed for the primary outcome | In a flow diagram, the number of participants:   - Evaluated for potential enrolment, if recorded - Excluded before randomisation with reasons:   - Not meeting the inclusion criteria   - Declined to participate   - Other reasons - Randomly assigned to each group - Who received intervention as allocated, by trial group - Who completed intervention as allocated, by trial group - Who completed follow-up as planned, by trial group - Included in the main analysis for the primary outcome, by trial group Where appropriate (e.g., nonpharmacologic interventions): - the number of care providers or centres performing the intervention in each group - the number of participants treated by each care provider or in each centre | Page 6 |

|  | 22b | For each group, losses and exclusions after randomisation, together with reasons | In a flow diagram, the number of participants:   - Lost to follow up, by trial group - Excluded from main analysis for the primary outcome, by trial group with reasons The wording “protocol deviation” is not sufficiently explicit and exact reasons should be reported | Page 6 |
| --- | --- | --- | --- | --- |
| Recruitment | 23a | Dates defining the periods of recruitment and follow-up for outcomes of benefits and harms | - Start and completion date of participant recruitment - Date when follow up ended - Duration of follow-up (e.g., median, interquartile range, minimum, maximum) | Page 6 |
|  | 23b | If relevant, why the trial ended or was stopped | If relevant,   - Reason for stopping the trial before completion as planned (e.g., result of an interim analysis, lack of funding, difficulty in recruiting patients) - Who made the decision to stop - Role of funder in decision to stop | N/A |
| Intervention and comparator delivery | 24a | Intervention and comparator as they were actually administered (e.g., where appropriate, who delivered the intervention/comparator, whether participants adhered, whether they  were delivered as intended [fidelity]) | - Who actually delivered the intervention/comparator (number and expertise) - How the intervention/comparator was actually administered - What intervention/comparator was actually administered - Participants’ adherence to the intervention/ comparator - Whether the intervention/comparator was delivered as intended (i.e., care provider’s   fidelity), where appropriate | Page 6 |
|  | 24b | Concomitant care received during the trial for each group | In each trial group:   - Number and percentage of participants receiving the different relevant concomitant interventions (i.e., interventions that could have affected the outcome) - Where relevant, the cumulative or average for each concomitant intervention taken over the trial period | Page 6 |
| Baseline data | 25 | A table showing baseline demographic and clinical characteristics for each group | Baseline characteristics for participants in each group in a table detailing:   - For continuous variables:   - Mean with standard deviation or median with percentiles (e.g., 25th, 75th), where appropriate - For binary and categorical variables:   - Numbers and percentages | Page 23 |
| Numbers analysed, | 26 | For each primary and secondary outcome, by group: | For all planned primary and secondary outcomes, in each group:   - The number of participants included in analysis - The number of participants with available data at the outcome time point | Page 6 |

| outcomes and estimation |  | - the number of participants included in the analysis - the number of participants with available data at the outcome time point - result for each group, and the estimated effect size and its precision (such as 95% confidence interval) - for binary outcomes, presentation of both absolute and relative effect size | - Reasons for missing data - For continuous outcomes:   - Summary of the outcome in each group (e.g., mean with standard deviation)   - Effect size (e.g., difference in means) and its precision (e.g., 95% confidence interval) - For binary or time-to-event outcomes:   - Summary of the outcome in each group (e.g., number of participants with the outcome event and denominator)   - Both absolute (e.g., risk difference, difference in median survival time, difference of restricted mean survival time) and relative effect size (e.g., risk ratio, odds ratio, hazard ratio, ratio of restricted mean survival time) and its   precision (e.g., 95% confidence interval) |  |
| --- | --- | --- | --- | --- |
| Harms | 27 | All harms or unintended events in each group | For each group preferably in a table with the number of participants at risk:   - Number of deaths - Number of participants withdrawn due to harms   For the systematically assessed harms, non-systematically assessed harms, and serious harms in each group preferably in a table with the number of participants at risk:   - Number of participants with at least one harm event - Number of events, if appropriate - If no adverse events were identified, this should be stated. - Where appropriate   - The estimated effect size and its precision (95% confidence interval)   - For binary outcomes/time-to-event outcomes, both absolute and relative   effects | Page 7 |
| Ancillary analyses | 28 | Any other analyses performed, including subgroup and sensitivity analyses, distinguishing pre-specified  from post-hoc | - Results for all other analyses performed - Describe which were pre-specified and which were post-hoc analyses | N/A |
| **Discussion** | | |  |  |
| Interpretation | 29 | Interpretation consistent with results, balancing benefits and harms, and considering other relevant evidence | - Brief summary of the trial results, balancing both benefits and harms of the intervention(s) - How the trial results relate to existing evidence (e.g. systematic review) - Avoid overinterpretation (‘spin’) | Page 9 |

| Limitations | 30 | Trial limitations, addressing sources of potential bias, imprecision, generalisability, and, if relevant,  multiplicity of analyses | - Any methodological limitations and, if relevant, any methods used to minimise or mitigate them - Any imprecision in the results - Generalisability of the results | Page 14 |
| --- | --- | --- | --- | --- |

Citation: Hopewell S, Chan AW, Collins GS, Hróbjartsson A, Moher D, Schulz KF, et al. CONSORT 2025 Statement: updated guideline for reporting randomised trials. BMJ. 2025; 388:e081123. <https://dx.doi.org/10.1136/bmj-2024-081123>

© 2025 Hopewell et al. This is an Open Access article distributed under the terms of the Creative Commons Attribution License (https://creativecommons.org/licenses/by/4.0/), which permits unrestricted use, distribution, and reproduction in any medium, provided the original work is properly cited.
